# Association of remnant cholesterol and newly diagnosed early‐onset type 2 diabetes mellitus in Chinese population: A retrospective cross‐sectional study

**DOI:** 10.1111/1753-0407.13498

**Published:** 2023-11-14

**Authors:** Wenjing Dong, Shiju Yan, Han Chen, Jian Zhao, Zengqiang Zhang, Weijun Gu

**Affiliations:** ^1^ Chinese PLA Medical College Beijing China; ^2^ Department of Endocrinology The First Medical Center of Chinese PLA General Hospital Beijing China; ^3^ Department of Gerontology Hainan Hospital of Chinese PLA General Hospital Sanya China; ^4^ Department of Orthopedics Hainan Hospital of Chinese PLA General Hospital Sanya China; ^5^ Department of Information Hainan Hospital of Chinese PLA General Hospital Sanya China

**Keywords:** dyslipidemia, early‐onset type 2 diabetes mellitus, insulin resistance, remnant cholesterol, risk factor

## Abstract

**Background:**

With the increasing incidence of diabetes worldwide, patients diagnosed with diabetes has been getting younger. Previous studies have shown that high remnant cholesterol (RC) level leads to an increased risk of cardiovascular disease events. However, the relationship between RC levels and newly diagnosed early‐onset type 2 diabetes mellitus (T2DM) is unknown. This study aimed to explore the association between RC and newly diagnosed early‐onset T2DM.

**Methods:**

A total of 606 patients newly diagnosed with early‐onset T2DM and 619 gender‐matched subjects with normal blood glucose levels were retrospectively enrolled in this study. All T2DM patients showed onset age of 18–40 years. Binary logistic regression analysis was performed to analyze independent risk factors and receiver operating characteristic (ROC) analysis was used to explore the predictive value of RC and other unconventional lipids. Moreover, the correlation between RC and insulin resistance in patients with newly diagnosed early‐onset T2DM was also examined with binary logistic regression analysis and Spearman correlation analysis.

**Results:**

Increased RC level was an independent risk factor for early‐onset T2DM (*p* < .05). The area under the curve on ROC analysis of RC was 0.805, 95% confidence interval (CI) was 0.781 ~ 0.826, sensitivity was 82.18% and specificity was 66.24%, which showed higher predictive value than those of triglyceride/high‐density lipoprotein cholesterol (TG/HDL‐C) ratio and total cholesterol (TC)/HDL‐C ratio. Cutoff value of RC was 0.32 mmol/L. Level of RC in early‐onset T2DM patients with moderate or severe insulin resistance was significantly higher than that in patients with mild insulin resistance (*p* < .0001). No difference in RC levels was found between patients with moderate and severe insulin resistance (*p* > .05). RC was still correlated with insulin resistance after adjusting the conventional lipid parameters (TG, TC, HDL‐C, and low‐density lipoprotein cholesterol) using partial correlation analysis.

**Conclusion:**

RC level was higher in patients with early‐onset T2DM and was correlated to the degree of insulin resistance as well. Patients aged 18–40 years with RC >0.32 mmol/L showed an increased risk of developing T2DM.

## Graphical abstract

**Highlights**

- This study was the first to explore the association between remnant cholesterol (RC) levels and newly diagnosed early‐onset type 2 diabetes mellitus (T2DM).
- We found that the risk of T2DM was increased when people aged 18–40 years had RC >0.32 mmol/L.
- RC level was correlated to the degree of insulin resistance.

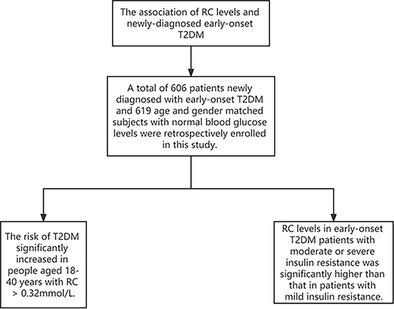

## 1 INTRODUCTION

With the development of social economy, change of lifestyle, and increase of obesity rate, the incidence of type 2 diabetes mellitus (T2DM) continues to rise rapidly and patients present a trend of getting younger worldwide, especially in developing countries, making diabetes the third largest cause of death.^1^, ^2^ According to the International Diabetes Federation,^3^ nearly 463 million people worldwide suffered from diabetes in 2019, accounting for 9.3% of global adult population. One Chinese study^4^ shows that the incidence of early‐onset diabetes mellitus (EODM) increased fourfold from 1997 to 2010 in China, and the number of patients is expected to increase by at least 20% in the next 20 years. Meanwhile, patients with EODM show a higher risk of microvascular and macrovascular diseases than those with late‐onset diabetes and type 1 diabetes mellitus at the same age, bringing great harm to patients, families, and society.^5^, ^6^, ^7^ Therefore, there is an urgent need for preventive screening and management of patients with early‐onset T2DM.

According to previous studies,^8^, ^9^ lipid abnormalities are risk factors for macrovascular disease as well as diabetes. In recent years, researchers have conducted a series of studies and explorations on the association of conventional and unconventional lipids with diabetes and its complications.^10^, ^11^ As one of classical unconventional lipids, remnant cholesterol (RC) is a type of triglyceride‐rich lipoprotein (TRL), consisting of intermediate density lipoprotein and very‐low‐density lipoprotein in fasting state or chylomicron remnants in nonfasting state, which is essentially cholesterol.^12^ The assessment of RC level is simple and convenient and RC level can be easily obtained with an established formula,^13^ which can provide valuable guidance for the management of relevant diseases. A large number of studies^14^, ^15^, ^16^ have shown that RC has a stronger atherogenic effect, which can significantly increase the risk of cardiovascular events and diabetic microvascular and macrovascular complications, due to a more direct effect of cholesterol content in TRLs than triglyceride (TG). Nevertheless, the relationship between RC level and the risk of early‐onset T2DM remains unclear. Therefore, this study explored the association of newly diagnosed early‐onset T2DM and RC, so as to provide evidence for early prevention and comprehensive management of early‐onset T2DM.

## 2 PATIENTS AND METHODS

### 2.1 Study subjects

This research involved 606 patients newly diagnosed with EODM in the First Medical Center and Hainan Hospital of PLA General Hospital from January 2012 to December 2022. The inclusion criteria were as follows: (1) the clinical diagnosis of T2DM following World Health Organization (1999) diagnostic criteria,^17^ (2) onset age was between 18 and 40 years and the duration of diabetes was < 1 year, and (3) no history of oral lipid‐lowering drugs taken in the past 3 months. Exclusion criteria were as follows: (1) type 1 diabetes mellitus, other special types of diabetes mellitus, or undetermined type of diabetes mellitus; (2) serious comorbidities such as heart, brain, and liver disorders or other malignant tumors; and (3) incomplete medical record or loss to follow‐up. The control group included subjects aged 18–40 years old with normal blood glucose (fasting blood glucose [FBG] < 7 mmol/L and postprandial blood glucose < 11.1 mmol/L) who underwent physical health check in the First Medical Center and Hainan Hospital of PLA General Hospital. After age and gender matching 619 subjects were included.

Data were manually abstracted by three investigators (D.W., Y.S., C.H.) from the electronic medical record database. This study was approved by the Ethics Committee of Chinese PLA General Hospital.

### 2.2 Definition

1. Drinking: men consumed >210 g per week and women >140 g per week.^18^
2. Smoking status: smoking for >6 months.
3. Family history of diabetes mellitus: history of DM in first‐degree relatives.
4. Nonalcoholic fatty liver disease: diagnosis criteria of nonalcoholic fatty liver disease.^19^
5. Hypertension: systolic blood pressure ≥140 mm Hg and/or diastolic blood pressure ≥90 mm Hg or hypertension that has been diagnosed and treated.

### 2.3 Study parameters

General data and biochemical parameters (gender, age of onset, initial symptoms, smoking status, drinking status, family history of DM, history of hypertension and fatty liver, height, weight, body mass index [BMI], waistline, hipline, and diabetic complications) were collected from the electronic medical record system.

Venous blood samples were collected from all patients after fasting overnight. Biochemical indicators such as FBG, fasting insulin (Fins), fasting C‐peptide (FCP), glycosylated hemoglobin (HbA1c), TG, total cholesterol (TC), high‐density lipoprotein cholesterol (HDL‐C), low‐density lipoprotein cholesterol (LDL‐C), urinary albumin‐to‐creatinine ratio, serum creatinine (Scr), uric acid (UA), alanine aminotransferase (ALT), aspartic transaminase (AST), gamma‐glutamyl transpeptidase (GGT), and white blood cell count (WBC) were measured by automatic biochemical analyzer in standard laboratory.

### 2.4 Calculation

Estimated glomerular filtration rate (eGFR) (mL/min per 1.73 m^2^) = 175* (Scr in mg/dL) −1.154*age (year) − 0.203* (0.742 for women).

Waist‐to‐hip ratio (WHR) = waistline (cm)/hipline (cm).

BMI (kg/m^2^) = weight (kg)/height^2^ (m).

RC (mmol/L) = TC (mmol/L)–HDL‐C (mmol/L)–LDL‐C (mmol/L).^20^

TG/HDL‐C ratio = TG (mmol/L)/HDL‐C (mmol/L).^21^

TC/HDL‐C ratio = TC (mmol/L)/HDL‐C (mmol/L).^21^

LDL/HDL‐C ratio = LDL‐C (mmol/L)/HDL‐C (mmol/L).^22^

Neutrophil to lymphocyte ratio (NLR) = neutrophil (10^9^/L)/lymphocyte (10^9^/L).

MLR = monocyte (10^9^/L)/ lymphocyte (10^9^/L).

PHR = platelet (10^9^/L)/HDL‐C (mmol/L).

### 2.5 Grouping

Homeostasis model assessment 2 (HOMA2) was used to evaluate insulin resistance (IR) in patients with EODM. With the help of HOMA2 calculator from www.Ocdem.ox.ac.uk, IR index (HOMA2‐IR) was calculated using FCP for patients treated with insulin and Fins for patients without insulin treatment. Early‐onset T2DM patients were divided into three groups, according to tertile of HOMA2‐IR value: mild IR group (<2.06, *N* = 336), moderate IR group (2.06 ~ 3.09, *N* = 170), and severe IR group (>3.09, *N* = 100).

### 2.6 Statistical analysis

Statistical analyzes were conducted using SPSS 26.0 (IBM SPSS, USA). Normally distributed data were described as mean ± SD. Independent samples *t* test or analysis of variance were used to analyze differences between groups. Asymmetrically distributed data were described as median and interquartile range M (QL, QU) and Mann–Whitney *U* test was used to analyze differences between groups. Categorical variables were described as numbers with percentages *n* (%) and compared with *χ* ^2^ test. Binary logistic regression analysis was used for multivariate analysis to determine the risk factors. A receiver operating characteristic (ROC) curve was subsequently drawn on the basis of predictive factors and sensitivity and specificity were evaluated using the area under the curve (AUC) value. The optimal cutoff value was determined by the Youden index. Spearman correlation and partial correlation analysis were used to analyze the relationship between RC and the degree of IR in patients with early‐onset T2DM. The link between RC level and other variables was also analyzed by Spearman correlation analysis. All analyzes used were two tailed with significance set at *p* < .05.

## 3 RESULTS

### 3.1 Baseline characteristics of study subjects

A total of 1225 subjects were enrolled, including 606 newly diagnosed with early‐onset T2DM patients and 619 healthy subjects with normal blood glucose. Mean onset age for all patients was 31.16 years ±0.16 SD with 950 (77.6%) male and 275 (22.4%) female. Compared with healthy individuals of parallel age, the proportion of drinking history, family history of diabetes, and fatty liver were higher in early‐onset T2DM patients (*p* < .0001). Moreover, the levels of systolic blood pressure (SBP), diastolic blood pressure (DBP), weight, BMI, WHR, FBG, HbA1c, TG, TC, LDL‐C, AST, ALT, GGT, WBC, PHR, MLR, neutrophil, monocyte, RC, TG/HDL‐C, TC/HDL‐C, and LDL‐C/HDL‐C in early‐onset T2DM patients were significantly higher whereas the levels of eGFR and HDL‐C were lower than those of control group (*p* < .001). In addition, no difference was found in other aspects (*p* > .05) (Table 1).

**TABLE 1:** Comparison of patients with and without early‐onset T2DM.

Variables	All (*n* = 1225)	Control group (*n* = 619)	Early‐onset T2DM group (*n* = 606)	*P* value
Gender (%)				.842
Male	950 (77.6)	482 (77.9)	468 (77.2)	
Female	275 (22.4)	137 (22.1)	138 (22.8)	
Onset age (year)	31.16 ± 0.16	31.06 ± 0.23	31.36 ± 0.24	.225
SBP (mm Hg)	122.09 ± 0.44	117.35 ± 0.54	126.93 ± 0.64	<.0001
DBP (mm Hg)	78.94 ± 0.34	76.49 ± 0.44	81.44 ± 0.49	<.0001
BMI (kg/m^2^)	26.15 ± 0.14	24.46 ± 0.16	27.88 ± 0.21	<.0001
Weight (kg)	76.88 ± 0.50	71.79 ± 0.60	82.07 ± 0.73	<.0001
Height (cm)	171.00 ± 0.26	170.86 ± 0.40	171.15 ± 0.32	.576
Smoking status (%)				.097
Yes	556 (45.4)	266 (43.0)	290 (47.9)	
No	669 (54.6)	353 (57.0)	316 (52.1)	
Drink (%)				<.0001
Yes	426 (34.8)	176 (28.4)	250 (41.3)	
No	799 (65.2)	443 (71.6)	356 (58.7)	
DM family history (%)				<.0001
Yes	355 (29.0)	25 (4.0)	330 (54.5)	
No	870 (71.0)	594 (96.0)	276 (45.5)	
FBG (mmol/L)	7.28 ± 0.11	4.86 ± 0.02	9.76 ± 0.17	<.0001
HbA1c (%)	7.48 ± 0.08	5.32 ± 0.01	9.68 ± 0.11	<.0001
Fatty liver (%)				<.0001
Yes	711 (58.0)	264 (42.6)	450 (74.3)	
No	513 (41.9)	355 (57.4)	156 (25.7)	
WHR	0.93 ± 0.23	0.89 ± 0.003	0.97 ± 0.003	<.0001
eGFR (ml/min/1.73 m^2^)	107.20 ± 0.77	120.10 ± 1.19	94.57 ± 0.63	<.0001
UA (umol/L)	370.57 ± 4.60	371.05 ± 3.80	370.09 ± 8.47	.918
TG (mmol/L)	1.67 (1.06, 2.64)	1.36 (0.88, 2.14)	2.04 (1.32, 3.23)	<.0001
TC (mmol/L)	4.80 ± 0.04	4.69 ± 0.04	4.91 ± 0.07	<.0001
HDL‐C (mmol/L)	1.09 ± 0.01	1.24 ± 0.01	0.93 ± 0.01	<.0001
LDL‐C (mmol/L)	3.03 ± 0.03	2.94 ± 0.04	3.11 ± 0.03	<.0001
ALT (U/L)	25.50 (14.90, 44.40)	21.90 (12.80, 36.30)	29.30 (18.00, 55.13)	<.0001
AST (U/L)	25.23 ± 0.62	22.18 ± 0.53	28.35 ± 1.11	<.0001
GGT (U/L)	46.35 ± 1.64	41.56 ± 1.80	51.23 ± 2.75	.003
WBC (10^9^/L)	6.77 ± 0.05	6.50 ± 0.07	7.05 ± 0.08	<.0001
Neutrophil (%)	0.55 ± 0.003	0.55 ± 0.003	0.54 ± 0.004	.609
NLR	1.71 ± 0.02	1.72 ± 0.03	1.70 ± 0.04	.709
Lymphocyte (%)	0.35 ± 0.002	0.34 ± 0.003	0.36 ± 0.004	<.0001
Monocyte (%)	0.07 ± 0.001	0.08 ± 0.001	0.06 ± 0.0008	<.0001
MLR	0.21 ± 0.003	0.23 ± 0.005	0.19 ± 0.004	<.0001
Platelet (10^9^/L)	250.52 ± 1.76	251.17 ± 2.11	249.85 ± 2.83	.708
PHR	253.59 ± 2.96	216.49 ± 2.99	291.49 ± 4.66	<.0001
RC (mmol/L)	0. 400.15,0.74)	0.20 (0.07,0.41)	0.64 (0.39,1.05)	<.0001
LDL‐C/HDL‐C	3.02 ± 0.03	2.69 ± 0.04	3.36 ± 0.05	<.0001
TG/HDL‐C	1.65 (0.87,2.91)	1.15 (0.62,2.04)	2.31 (1.41,4.04)	<.0001
TC/HDL‐C	4.88 ± 0.07	4.03 ± 0.05	5.76 ± 0.12	<.0001

Abbreviations: ALT, alanine aminotransferase; AST, aspartic transaminase; BMI, body mass index; DBP, diastolic blood pressure; eGFR, estimated glomerular filtration rate; FBG, fasting blood‐glucose; GGT, gamma‐glutamyl transpeptidase; HbA1c, glycosylated hemoglobin; HDL‐C, high‐density lipoprotein cholesterol; LDL‐C, low‐density lipoprotein cholesterol; LDL‐C/HDL‐C, LDL‐C/HDL‐C ratio; MLR, monocyte to lymphocyte ratio; NLR, neutrophil to lymphocyte ratio; PHR, platelet/HDL‐C ratio; RC, remnant cholesterol; SBP, systolic blood pressure; T2DM, type 2 diabetes mellitus; TC, total cholesterol; TC/HDL‐C, TC/HDL‐C ratio; TG, triglyceride; TG/HDL‐C, TG/HDL‐C ratio; UA, uric acid; WBC, white blood cell; WHR, waist‐to‐hip ratio.

### 3.2 Binary logistic regression analysis and predictive value of RC for early‐onset T2DM occurrence

**FIGURE 1:**
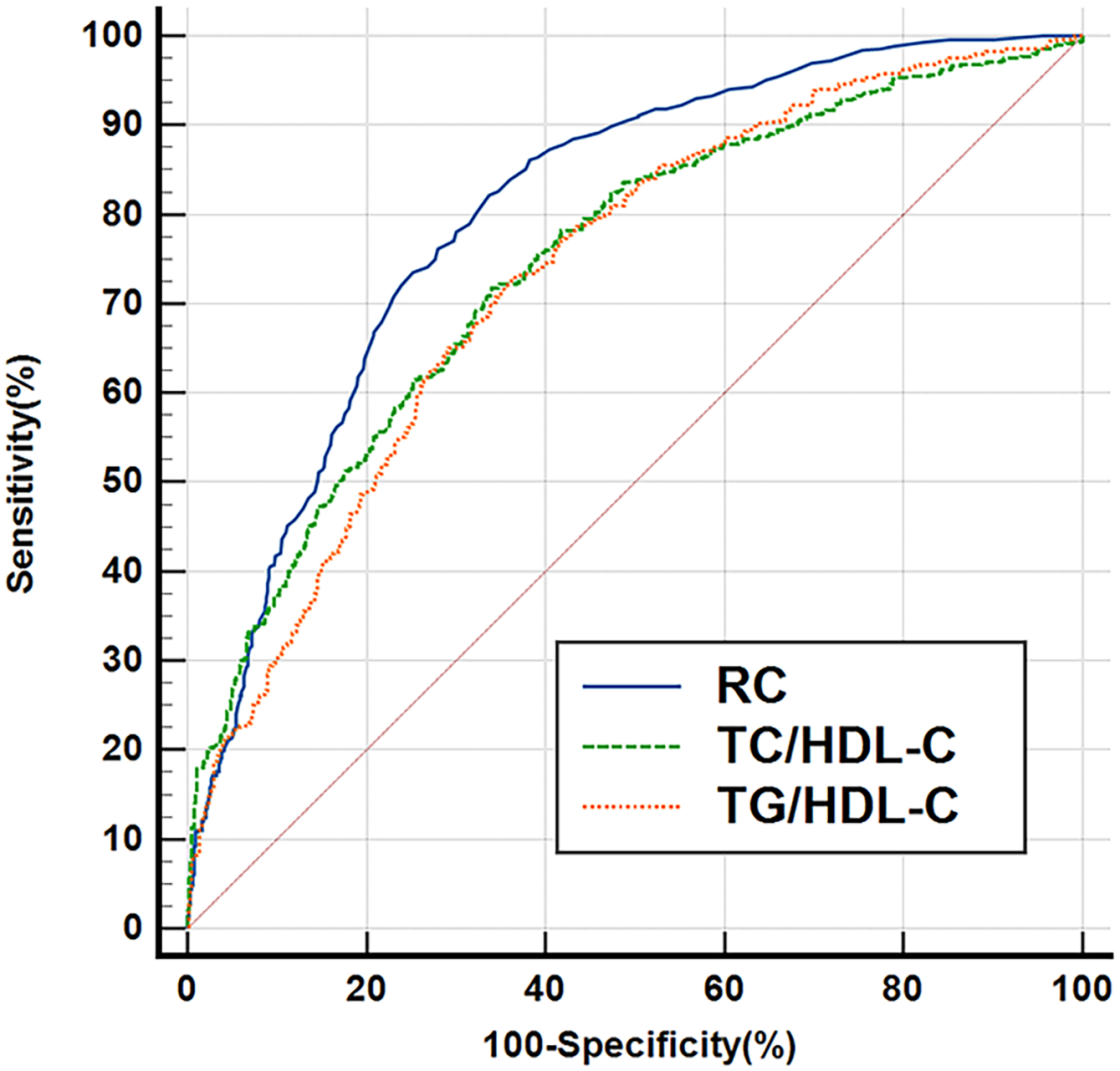
Receiver operating characteristic (ROC) curves of remnant cholesterol (RC), TG/HDL‐C, and TC/HDL‐C. RC had higher predictive value than TG/HDL‐C or TC/HDL‐C (AUC = 0.805, 95% CI: 0.781–0.826, *p* < .001, cutoff: 0.32, sensitivity: 82.18%, specificity: 66.24%). AUC, area under the curve; HDL‐C, high‐density lipoprotein cholesterol; TC, total cholesterol; TG, triglyceride.

Those variables that were statistically significant in the univariate analysis while not interfering with each other were included in the binary regression analysis model. The results demonstrated that RC, BMI, WHR, family history of DM, MLR, PHR, eGFR, TG/HDL‐C, TC/HDL‐C, SBP, and DBP were independently associated with the occurrence of early‐onset T2DM (*p* < .05) (Table 2). To further explore the predictive value of RC and other unconventional lipids, ROC curves were drawn and demonstrated an AUC of 0.805 (95% CI: 0.781–0.826, *p* < .001) for RC, showing that RC had higher predictive value than any other factors (Figure 1 **)**.

**TABLE 2:** Multivariate analysis of patients with early‐onset T2DM.

Variables	*r*	SE	*p*	(β) 95% CI
BMI (kg/m^2^)	−0.082	0.032	0.012	(0.865 ~ 0.982)
DM family history	2.957	0.286	<0.0001	(10.9838 ~ 33.692)
Fatty liver	0.069	0.248	0.962	(0.659 ~ 1.740)
MLR	−8.270	1.237	<0.0001	(0.0001 ~ 0.003)
PHR	0.004	0.001	0.001	(1.002 ~ 1.007)
ALT (U/L)	0.003	0.003	0.285	(0.999 ~ 1.010)
AST (U/L)	0.005	0.007	0.483	(0.991 ~ 1.019)
eGFR (ml/min/1.73 m^2^)	0.063	0.006	<0.0001	(1.053 ~ 1.077)
TC/HDL‐C	0.185	0.099	0.042	(0.991 ~ 1.460)
TG/HDL‐C	−0.201	0.057	<0.0001	(0.731 ~ 0.915)
RC (mmol/L)	1.269	0.275	<0.0001	(2.076 ~ 3.092)
WHR	0.179	0.023	<0.0001	(1.143 ~ 1.251)
SBP (mm Hg)	0.050	0.012	<0.0001	(1.028 ~ 1.076)
DBP (mm Hg)	0.054	0.014	<0.0001	(0.922 ~ 0.974)

Abbreviations: ALT, alanine aminotransferase; AST, aspartic transaminase; BMI, body mass index; CI, confidence interval; DBP, diastolic blood pressure; DM, diabetes mellitus; eGFR, estimated glomerular filtration rate; HDL‐C, high‐density lipoprotein cholesterol; MLR, monocyte to lymphocyte ratio; PHR, platelet/HDL‐C ratio; RC, remnant cholesterol; SBP, systolic blood pressure; T2DM, type 2 diabetes mellitus; TC/HDL‐C, TC/HDL‐C ratio; TG/HDL‐C, TG/HDL‐C ratio; WHR, waist‐to‐hip ratio.

### 3.3 Baseline characteristics of patients with early‐onset T2DM: with reference to insulin resistance degree

**FIGURE 2:**
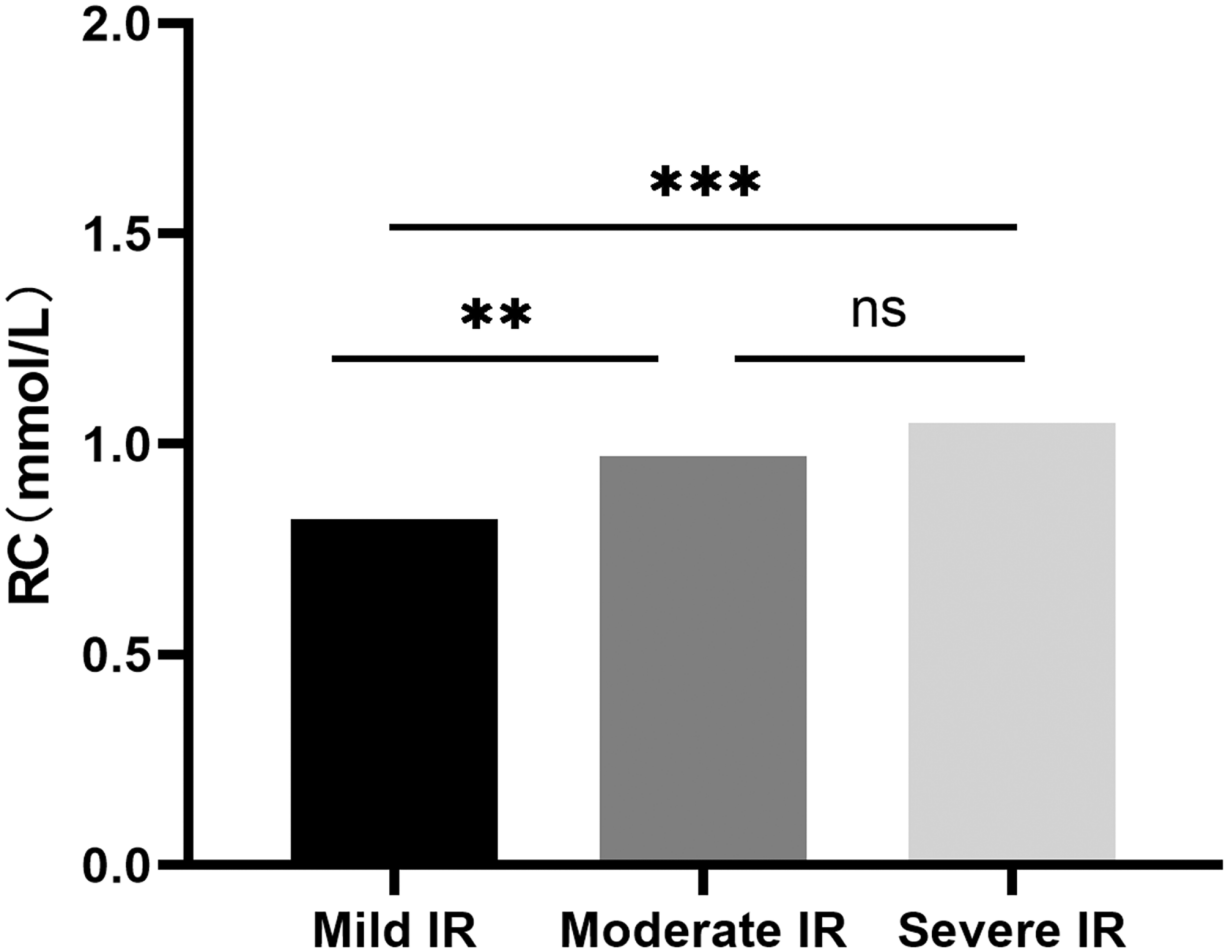
Remnant cholesterol (RC) levels in different insulin resistance (IR) groups. RC levels in mild IR group was significantly lower than that in moderate (*p* < .01) or severe IR group (*p* < .001) and no difference was found in RC level between moderate and severe IR groups (*p* > .05). **p < .01, ***p < .001.

Of all patients, 336 (55.4%) were in the mild IR group, 170 (28.1%) were in the moderate IR group, and 100 (16.5%) were in the severe IR group, with an average onset age of 31.36 years ±0.32 SD, 31.54 years ±0.45 SD and 31.39 years ±0.58 SD respectively. As the degree of insulin resistance increased, the proportion of patients with diabetic nephropathy and history of fatty liver, level of SBP, DBP, BMI, FBG, weight, WHR, UA, TG, TC, AST, ALT, GGT, WBC, PHR, TG/HDL‐C, TC/HDL‐C, and LDL‐C/HDL‐C in early‐onset T2DM showed an upward trend (*p* < .05). The level of eGFR and HDL‐C presented a decreasing trend (*p* < .05). Furthermore, level of RC in the mild IR group was significantly lower than that in the moderate and severe IR group (*p* < .01), whereas there was no difference in RC level between the moderate and severe IR groups (*p* > .05). Three groups did not differ in other parameters (*p* > .05) (Table 3, Figure 2).

**TABLE 3:** Comparison of early‐onset T2DM patients with different insulin resistance degree.

Variables	Mild IR group	Moderate IR group	Severe IR group	*P* value
Gender (%)				.793
Male	258 (76.8)	130 (76.5)	80 (80.0)	
Female	78 (23.2)	40 (23.5)	20 (20.0)	
Onset age (year)	31.36 ± 0.32	31.54 ± 0.45	31.39 ± 0.58	.884
SBP (mm Hg)	124.36 ± 0.83	129.70 ± 1.26	131.58 ± 1.68	<.0001
DBP (mm Hg)	79.43 ± 0.63	84.03 ± 1.02	84.62 ± 1.21	<.0001
BMI (kg/m^2^)	26.30 ± 0.25	29.18 ± 0.42	30.94 ± 0.56	<.0001
Weight (kg)	77.16 ± 0.87	86.21 ± 1.42	91.47 ± 2.00	<.0001
Height (cm)	170.95 ± 0.46	171.58 ± 0.62	171.36 ± 0.72	.627
Smoking status (%)				.335
No	166 (49.4)	95 (55.9)	55 (55.0)	
Yes	170 (50.6)	75 (44.1)	45 (45.0)	
Drink (%)				.466
No	198 (58.9)	104 (61.2)	54 (54.0)	
Yes	138 (41.1)	66 (38.8)	46 (46.0)	
DM family history (%)				.093
No	166 (49.4)	72 (42.4)	38 (38.0)	
Yes	170 (51.7)	98 (57.6)	62 (62.0)	
FBG (mmol/L)	9.24 ± 0.22	10.41 ± 0.33	10.81 ± 0.49	<.0001
HbA1c (%)	9.69 ± 0.16	9.65 ± 0.17	9.74 ± 0.23	.996
Fatty liver (%)				<.0001
No	126 (37.5)	19 (11.2)	11 (11.0)	
Yes	210 (62.5)	151 (88.8)	89 (89.0)	
Diabetic retinopathy (%)				.790
No	317 (94.3)	160 (94.1)	96 (96.0)	
Yes	19 (5.7)	10 (5.9)	4 (4.0)	
DPN (%)				.993
No	319 (94.9)	161 (94.7)	94 (94.0)	
Yes	17 (5.1)	9 (5.3)	6 (6.0)	
Macrovascular diseases (%)				.563
No	267 (79.5)	128 (75.3)	78 (78.0)	
Yes	69 (20.5)	42 (24.7)	22 (22.0)	
Diabetic nephropathy (%)				.016
No	321 (95.5)	155 (91.2)	88 (88.9)	
Yes	15 (4.5)	15 (8.8)	12 (12.1)	
WHR	0.95 ± 0.004	0.98 ± 0.005	0.99 ± 0.006	<.0001
eGFR (ml/min/1.73 m^2^)	122.98 ± 1.80	118.17 ± 1.99	115.26 ± 2.58	.046
UA (umol/L)	348.26 ± 11.34	375.26 ± 8.84	437.30 ± 32.54	.001
TG (mmol/L)	2.56 ± 0.18	3.56 ± 0.25	4.17 ± 0.11	<.0001
TC (mmol/L)	4.76 ± 0.11	5.01 ± 0.11	5.20 ± 0.22	.063
HDL‐C (mmol/L)	0.97 ± 0.02	0.90 ± 0.02	0.85 ± 0.05	.02
LDL‐C (mmol/L)	2.88 ± 0.05	2.97 ± 0.08	3.00 ± 0.11	.496
ALT (U/L)	24.30 (14.45,41.10)	37.50 (23.00,61.20)	54.25 (28.38,95.70)	<.0001
AST (U/L)	16.80 (12.75,25.95)	22.20 (16.00,32.80)	28.85 (18.45,48.70)	<.0001
GGT (U/L)	44.34 ± 4.30	52.99 ± 3.56	72.22 ± 6.82	.001
WBC (10^9^/L)	6.81 ± 0.10	7.23 ± 0.13	7.45 ± 0.22	.003
Neutrophil (%)	0.54 ± 0.005	0.55 ± 0.007	0.54 ± 0.009	.901
NLR	1.67 ± 0.05	1.71 ± 0.07	1.69 ± 0.09	.970
Lymphocyte (%)	0.36 ± 0.005	0.36 ± 0.006	0.36 ± 0.009	.765
Monocyte (%)	0.06 ± 0.001	0.07 ± 0.001	0.07 ± 0.002	.920
MLR	0.19 ± 0.005	0.19 ± 0.006	0.19 ± 0.009	.983
Platelet (10^9^/L)	248.38 ± 3.8	247.47 ± 5.18	259.45 ± 7.61	.346
PHR	280.53 ± 5.98	294.36 ± 9.05	322.50 ± 13.08	.005
RC (mmol/L)	0.52 (0.33,0.84)	0.78 (0.48,1.23)	0.78 (0.52,1.67)	<.0001
LDL‐C/HDL‐C	3.23 ± 0.07	3.46 ± 0.09	3.59 ± 0.13	.02
TG/HDL‐C	1.93 (1.13,3.02)	2.81 (1.82,4.87)	2.84 (1.99,7.04)	<.0001
TC/HDL‐C	5.36 ± 0.15	6.00 ± 0.19	6.59 ± 0.36	<.0001
UACR	8.80 (5.30,15.00)	9.00 (5.00,18.30)	11.00 (6.33,25.75)	.075

Abbreviations: ALT, alanine aminotransferase; AST, aspartic transaminase; BMI, body mass index; DBP, diastolic blood pressure; DM, diabetes mellitus; DPN, diabetic peripheral neuropathy; eGFR, estimated glomerular filtration rate; FBG, fasting blood‐glucose; GGT, gamma‐glutamyl transpeptidase; HbA1c, glycosylated hemoglobin; HDL‐C, high‐density lipoprotein cholesterol; IR, insulin resistance; LDL‐C, low‐density lipoprotein cholesterol; LDL‐C/HDL‐C, LDL‐C/HDL‐C ratio; MLR, monocyte to lymphocyte ratio; NLR, neutrophil to lymphocyte ratio; PHR, platelet/HDL‐C ratio; RC, remnant cholesterol; SBP, systolic blood pressure; T2DM, type 2 diabetes mellitus; TC, total cholesterol; TC/HDL‐C, TC/HDL‐C ratio; TG, triglyceride; TG/HDL‐C, TG/HDL‐C ratio; UA, uric acid; UACR, urinary albumin‐to‐creatinine ratio; WBC, white blood cell; WHR, waist‐to‐hip ratio.

### 3.4 Correlation between RC and other variables in the lipid profiles with degrees of IR

Spearman correlation analysis was used to evaluate the correlation between variables in the lipid profile (RC, TG, TC, HDL‐C, LDL‐C, TG/HDL‐C, TC/HDL‐C, LDL‐C/HDL‐C) and the degree of IR. Results displayed that all lipid parameters were correlated with IR degrees (*p* < .001), except for LDL‐C. After adjusting the conventional lipid parameters (TG, TC, HDL‐C, LDL‐C) using partial correlation analysis, RC was still correlated with insulin resistance (*p* < .05) (Table 4).

**TABLE 4:** The comparison between lipid profiles and IR degree in early‐onset T2DM.

Variables	Spearman correlation analysis		Partial correlation analysis	
	*r*	*p* value	*r*	*p* value
RC (mmol/L)	0.271	<.0001	‐	‐
TC (mmol/L)	0.185	<.0001	0.067^a^	.048
HDL‐L (mmol/L)	−0.13	.001	0.093^b^	.022
LDL‐L (mmol/L)	0.033	.42	0.116^c^	.004
TG (mmol/L)	0.312	<.0001	−0.096^d^	.018
TG/HDL‐L	0.298	<.0001	−0.024^e^	.556
TC/HDL‐L	0.238	<.0001	−0.027^f^	.507
LDL‐L/HDL	0.124	.002	0.099^g^	.015

*Note*: Associations between lipid profiles and IR degree by Spearman correlation analysis and the association between RC and IR degree by partial correlation analysis ^a^Adjusted for TC; ^b^Adjusted for HDL‐L; ^c^Adjusted for LDL‐L; ^d^Adjusted for TG; ^e^Adjusted for TG/HDL‐L; ^f^Adjusted for TC/HDL‐L; ^g^Adjusted for LDL‐L/HDL.

Abbreviations: HDL‐C, High‐density lipoprotein cholesterol; IR, insulin resistance; LDL‐C, Low‐density lipoprotein cholesterol; LDL‐C/HDL‐C, LDL‐C/HDL‐C ratio; RC, remnant cholesterol; T2DM, type 2 diabetes mellitus; TC, total cholesterol; TC/HDL‐C, TC/HDL‐C ratio; TG, triglyceride; TG/HDL‐C, TG/HDL‐C ratio.

### 3.5 Association of RC level and other potential risk factors of early‐onset T2DM

We evaluated the link between RC and other variables in early‐onset T2DM patients with Spearman correlation analysis. It suggested that RC had a significant and positive correlation with BMI (*r* = 0.107), WHR (*r* = 0.159), fatty liver (*r* = 0.297), TC (*r* = 0.815), TG (*r* = 0.849), TG/HDL‐C (*r* = 0.835), TC/HDL‐C (*r* = 0.703), LDL‐C /HDL‐C (*r* = 0.281), UA (*r* = 0.250), PHR (*r* = 0.289), SBP (*r* = 0.107), DBP (*r* = 0.081) (all *p* < .05); RC had a significant and negative correlation with HDL‐C (*r* = 0.‐0.434, *p* < .0001) (Table 5).

**TABLE 5:** The correlation of RC with other potential risk factors in early‐onset T2DM.

Variables	Spearman correlation analysis	
	*r*	*p* value
SBP (mm Hg)	0.107	.008
DBP (mm Hg)	0.081	.046
BMI (kg/m^2^)	0.107	<.0001
FBG (mmol/L)	0.077	.058
WHR	0.159	<.0001
Fatty liver	0.297	<.0001
Initial ketosis at onset	−0.027	.509
Weight loss at onset	0.034	.407
eGFR (ml/min/1.73 m^2^)	−0.032	.431
UA (umol/L)	0.25	<.0001
TG (mmol/L)	0.849	<.0001
TC (mmol/L)	0.815	<.0001
HDL‐L (mmol/L)	−0.434	<.0001
LDL‐C (mmol/L)	−0.003	.944
TG/HDL‐L	0.835	<.0001
TC/HDL‐C	0.703	<.0001
LDL‐C/HDL‐C	0.281	<.0001
PHR	0.289	<.0001
MLR	0.008	.844
NLR	0.012	.767

Abbreviations: BMI, body mass index; DBP, diastolic blood pressure; eGFR, estimated glomerular filtration rate; FBG, fasting blood‐glucose; HDL‐C, high‐density lipoprotein cholesterol; LDL‐C, low‐density lipoprotein cholesterol; LDL‐C/HDL‐C, LDL‐C/HDL‐C ratio; MLR, monocyte to lymphocyte ratio; NLR, neutrophil to lymphocyte ratio; PHR, platelet/HDL‐C ratio; RC, remnant cholesterol; SBP, systolic blood pressure; T2DM, type 2 diabetes mellitus; TC, total cholesterol; TC/HDL‐C, TC/HDL‐C ratio; TG, triglyceride; TG/HDL‐C, TG/HDL‐C ratio; UA, uric acid; WHR, waist‐to‐hip ratio.

## 4 DISCUSSION

DM is a kind of chronic metabolic disease with disorders of carbohydrate, fat, and protein metabolism, which is caused by the interaction of genetic and acquired factors. Previous studies^23^ have reported that T2DM have complex lipid metabolism disorders, which participate in DM progression from beginning. Conventional lipids, namely high TG and low HDL‐C levels, are known to be associated with an increased risk of DM. In this study, we noted that indicators such as RC, TG/HDL‐C, TC/HDL‐C, BMI, WHR, blood pressure levels, and family history of diabetes were independently associated with the risk of early‐onset T2DM, which was basically consistent with the study of Yu et al.^9^ As we know, manifestations of metabolic syndromes (central obesity, dyslipidemia, and hypertension) are closely related to the pathogenesis and development of T2DM. Meanwhile, as a genetic predisposition disease, T2DM is often characterized by familial aggregation, which might be related to gene mutations that damage islet B cells.^24^ Therefore, people with obesity, hyperlipidemia, hypertension, and family history of DM should be actively educated to change their lifestyles, take more physical exercises, perform oral glucose tolerance test, and monitor blood pressure, blood lipid, and blood glucose regularly to avoid diabetes.

Recently, many studies have found the association of unconventional lipid parameters such as TG/HDL‐C and TC/HDL‐C with the incidence of DM. Studies^10^ have reported that high RC level is positively correlated with the occurrence and severity of retinopathy in patients with T2DM. Wang et al^25^ also find that RC could increase the risk of cardiovascular disease, especially in patients with T2DM. Sheng et al^11^ suggest that unconventional lipid parameters have higher diagnostic performance for future diabetes risk than conventional lipid parameters and both TG/HDL‐C and TC/HDL‐C are great indicators for predicting the occurrence of diabetes. A cohort study^26^ based on 1243 adults in the United States shows that TG/HDL‐C is one of the independent risk factors for DM, and it could better predict the risk of diabetes in women in the future. In addition, Seo's study^27^ displays that TC/HDL‐C is independently associated with the risk of type 2 diabetes. In our study, we found that RC was better than TG/HDL‐C and TC/HDL‐C in predicting the risk of type 2 diabetes in young adults aged 18–40 years and the cutoff value of RC was 0.32 mmol/L. Because TRLs in RC contain more and higher molecular weight cholesterol, we hypothesized that the damage to islet β cells caused by cholesterol toxicity might be an important reason for the increased risk of early‐onset T2DM. Many experiments^28^ have shown that when exposed to high cholesterol environment, pancreatic β cells would significantly reduce the insulin secretion caused by glucose stimulation, and it is believed that high cholesterol might inhibit glucose metabolism by affecting the translocation and activation of glucokinase. Furthermore, high cholesterol is observed mainly to be concentrated in insulin granules in β cells. Excess cholesterol accumulation in insulin granules not only influences the structure of membrane proteins, but also inhibits their movement on the plasma membrane.^29^, ^30^ In addition, high cholesterol level also suppresses β cells proliferation by inducing endoplasmic reticulum and mitochondrial dysfunction.^31^, ^32^ As a consequence, we should focus more on young people with high RC level and early screening for diabetes actively.

We all know that insulin resistance and pancreatic β‐cells dysfunction are two vital mechanisms in the development of T2DM. Latest survey^33^ finds that insulin resistance has become the main cause of early‐onset T2DM with the increasing prevalence of obesity and overweight in young people. Studies^34^ also indicate that IR and visceral fat accumulation causing glucose and lipid metabolism disorders are important incentives of metabolic diseases and play a crucial role in the pathogenesis and development of early‐onset T2DM. Likewise, our results found that RC level was correlated to the IR degree in patients with early‐onset T2DM. What is more, RC level in patients in the mild IR group was significantly lower than that in patients in the moderate or severe IR groups, whereas no difference was found in the level of RC in patients with moderate and severe insulin resistance. This implied that IR could be another mechanism of RC‐induced early‐onset T2DM and play a dominant effect in the onset stage rather than progression phase of early‐onset T2DM. Previous studies^35^, ^36^ have also found that RC is closely associated with IR and RC was speculated to play an essential role in the relation of insulin resistance, hypertriglyceridemia, and atherosclerosis. A randomized controlled trial in Japan^37^ suggests that patients in the empagliflozin group have significantly lower RC levels than those in placebo group, which is attributed to the improved insulin sensitivity in diabetic patients with IR. In our study, RC was still correlated with insulin resistance, even after adjusting for the influence of conventional lipid factors using partial correlation analysis. Meanwhile, many studies^38^ have found that IR could also interfere with RC metabolism. IR in the liver could inhibit the activity of TRLs in cells, which leads to reduced clearance of TRLs and continuous accumulation in human body.^38^ Consequently, RC and IR might have a causal relationship with each other, and the specific connection and mechanism need to be verified by large‐scale experiments and studies in the future.

RC could also trigger low‐grade systemic inflammation and we inferred whether inflammation participates in and mediates the association between RC and newly onset diabetes.^13^ Previous human and animal experimental studies^39^, ^40^, ^41^ have shown that TRLs enriched in RC could easily enter the arterial intima, not only taken up by macrophages in the intima and causing local intimal inflammation but also causing inflammation and necrosis of pancreatic cells through toxic free fatty acids and monoacylglycerol formed after hydrolysis. NLR and MLR are novel and widely accepted inflammatory markers, which have been identified to be related with the severity and prognosis of many cancers and cardiovascular and psychological diseases.^42^, ^43^, ^44^ Dr. Jialal^45^ finds that PHR could better predict metabolic syndromes and have potential to become a new inflammatory marker for atherosclerosis. In our study, Spearman correlation analysis was used to find out that only PHR was significantly correlated with RC (*r* = 0.289, *p* < .0001), which was consistent with findings of Song.^46^ The association of inflammation and RC still needs to be further researched. UA has the effect of promoting oxidation and inflammation, which could cause microvascular injury by stimulating the renin‐angiotensin system, inhibiting the proliferation of endothelial nitric oxide, and participate in metabolic syndrome.^47^, ^48^ Numerous studies^48^, ^49^ have shown that hyperuricemia is an important risk factor for many diseases such as cardiovascular disease, chronic kidney disease, and T2DM. Similarly, a community‐based cross‐sectional study^50^ in China finds a close association of dyslipidemia and hyperuricemia. Our results also showed a significant correlation between UA and RC (*r* = 0.25, *p* < .0001). Whether urate‐lowering therapy could reduce RC level and improve IR needs more in‐depth research.

However, our study faced several certain limitations. First, our study was a double‐center retrospective cross‐sectional study, which could not well determine the causal relationship between early‐onset T2DM and RC, and the sample size was relatively small. Second, we mainly studied the link between fasting RC level and T2DM in Chinese young people aged 18–40 years but the association of the RC level of different races, nonfasting RC level, and different types of diabetes needs more investigation. Lastly, molecular mechanisms underlying these relationships should be further explored.

To our known, this study is the first to explore the association between RC levels and newly diagnosed early‐onset T2DM. We found that the risk of T2DM was increased when people aged 18–40 years had RC >0.32 mmol/L and RC level was related to IR degree. To be specific, RC level in patients with mild IR was significantly lower than that in patients with moderate or severe IR. Clinicians could screen the high‐risk population of early‐onset T2DM by detecting RC level combined with traditional diabetes risk factors, so as to offer more scientific and reasonable management of patients.

## AUTHOR CONTRIBUTIONS

Weijun Gu, Wenjing Dong, and Shiju Yan designed the concept of the study. Wenjing Dong, Han Chen, Jian Zhao, and Shiju Yan collected and analyzed the data. Wenjing Dong, Zengqiang Zhang, and Weijun Gu critically revised the analysis. Shiju Yan and Wenjing Dong wrote the draft manuscript and Weijun Gu revised the draft manuscript. All authors listed have made a substantial, direct, and intellectual contribution to the work and approved it for publication.

## FUNDING INFORMATION

This study was supported by Hainan Province Clinical Medical Center.

## DISCLOSURE

The authors declare that the research was conducted in the absence of any commercial or financial relationships that could be construed as a potential conflict of interest.

## Data Availability

The raw data supporting the conclusions of this article will be made available from the corresponding author upon request and without undue reservation.
